# Molecular Therapies for Choroideremia

**DOI:** 10.3390/genes10100738

**Published:** 2019-09-23

**Authors:** Jasmina Cehajic Kapetanovic, Alun R. Barnard, Robert E. MacLaren

**Affiliations:** 1 Nuffield Laboratory of Ophthalmology, University of Oxford, Oxford OX3 9DU, UK; alun.barnard@eye.ox.ac.uk (A.R.B.); robert.maclaren@eye.ox.ac.uk (R.E.M.); 2 Oxford Eye Hospital, Oxford University Hospitals NHS Foundation Trust, Oxford OX3 9DU, UK

**Keywords:** choroideremia, gene therapy, REP1, inherited retinal disease, treatment

## Abstract

Advances in molecular research have culminated in the development of novel gene-based therapies for inherited retinal diseases. We have recently witnessed several groundbreaking clinical studies that ultimately led to approval of Luxturna, the first gene therapy for an inherited retinal disease. In parallel, international research community has been engaged in conducting gene therapy trials for another more common inherited retinal disease known as choroideremia and with phase III clinical trials now underway, approval of this therapy is poised to follow suit. This chapter discusses new insights into clinical phenotyping and molecular genetic testing in choroideremia with review of molecular mechanisms implicated in its pathogenesis. We provide an update on current gene therapy trials and discuss potential inclusion of female carries in future clinical studies. Alternative molecular therapies are discussed including suitability of *CRISPR* gene editing, small molecule nonsense suppression therapy and vision restoration strategies in late stage choroideremia.

## 1. Introduction

Choroideremia is a rare X-linked recessive inherited retinal disease caused by sequence variations or deletions in the *CHM* gene which are usually functionally null mutations, leading to deficiency in Rab escort protein 1 (REP1) [1,2,3]. The estimated prevalence is 1 in 50,000 males. Although REP1 is expressed ubiquitously, in humans choroideremia appears only to affect the retinal pigment epithelium (RPE) layer of the eye, leading to a characteristic clinical phenotype of progressive centripetal retinal degeneration. In Ancient Greek, the name choroid derives from *χόριον* (khórion, “skin”) and *εἶδος* (eîdos, “resembling”). The suffix ‘eremia’ (*ἐρημία*) was added to describe the barren appearance (from the root word meaning wasteland or desert). Hence the literal translation of choroideremia is, in relation to the eye, ‘the skin-resembling part is deserted’. Interestingly, the incorrect spelling ‘*choroideraemia’* has been used previously, but this may be based on misinterpretation of the suffix being derived from *αἷμα* (haima, blood), into which the ‘ae’ diphthong is still substituted in many non-US English usages. Importantly however, despite reference to the choroid in the name, the disease is now known to be driven primarily by the loss of the RPE, followed by the secondary degeneration of photoreceptors and choroidal atrophy [4]. Recent evidence has shed light on the molecular mechanisms of REP1 contribution to retinal degeneration in choroideremia, describing its essential role in post-translational modification of proteins and in intracellular trafficking of molecules [5]. The process affects primarily the RPE and pigment clumping is the first sign, long before photoreceptor loss. However, since the RPE has an essential role in retinal isomerization in the visual cycle which is more important for rod compared to cone function and hence rod function is impaired quite early in the disease process. As a result, the disease presents with early childhood nyctalopia, but the majority of patients retain excellent visual acuity until the very end stages of disease, presumably because Müller cells can still contribute to the cone visual cycle in the absence of RPE [6,7,8].

In this chapter we review advances in molecular therapies that have resulted in the development of adeno-associated vector (AAV) gene replacement therapy for choroideremia. The therapy is currently being explored in multiple clinical trials worldwide, having recently reached phase III in the development (Table 1). We discuss new insights into the clinical phenotyping and genotyping of choroideremia male patients and female carriers, including progress from the natural history studies, that will aid disease characterisation, monitoring of disease progression and interpretation of clinical trial endpoints. The review discusses current knowledge and progress in molecular mechanisms of choroideremia and the development of emerging potential therapies. 

## 2. Choroideremia Phenotype

**Figure 1:**
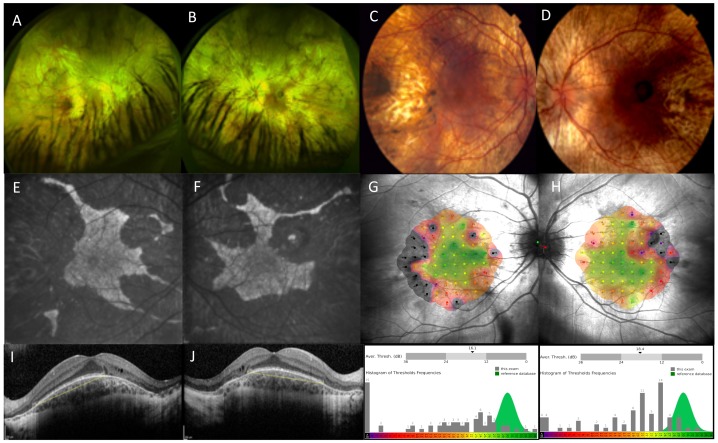
Retinal imaging in choroideremia. Widefield optomaps, Optos, Dumfernline, UK (**A**,**B**) and Heidelberg Spectralis imaging, Heidelberg, Germany (**C**–**F**) showing choroideremia phenotype in an affected male. Colour fundus photographs (**C**,**D**) show extensive retinal degeneration with choroidal atrophy and visualisation of underlying pale sclera. Fundus autofluorescence (**E**,**F**) shows typical patterns of sharply demarcated areas of remaining tissue (hyperfluorescent) against atrophic retina (hypofluorescent background). Mesopic microperimetry, MAIA CenterVue SpA, Padova, Italy (**G**,**H**) measures central retinal sensitivity that closely maps areas of residual retina as seen on autofluorescence. Sensitivity maps are shown with corresponding histograms of threshold frequencies. Spectral domain optical coherence tomography, Heidelberg, Germany (**I**,**J**) shows retinal structure in cross-section with distribution of ellipsoid zone (yellow line) and preserved inner retinal layers.

Choroideremia manifests with a pathognomonic fundus appearance characterised by progressive degeneration of retina and choroid (Figure 1). The degeneration starts in a ring around the mid-periphery of the retina and expands both centripetally towards the fovea and anteriorly to the pars plana [7,15,16]. The anatomical changes are accompanied by loss of functional scotopic vision and the reduction of the mid-peripheral visual field that begins during the first and second decade of life. The visual acuity is generally well preserved until late in the disease process, usually until the fifth decade of life, when the degeneration starts to encroach onto the fovea [6,7,8,15,16].

It remains somewhat unclear whether the RPE, the retina and the choroid are all primarily affected, or whether one or more of these tissues is secondarily affected during the pathogenesis of choroideremia [4].

**Figure 2:**
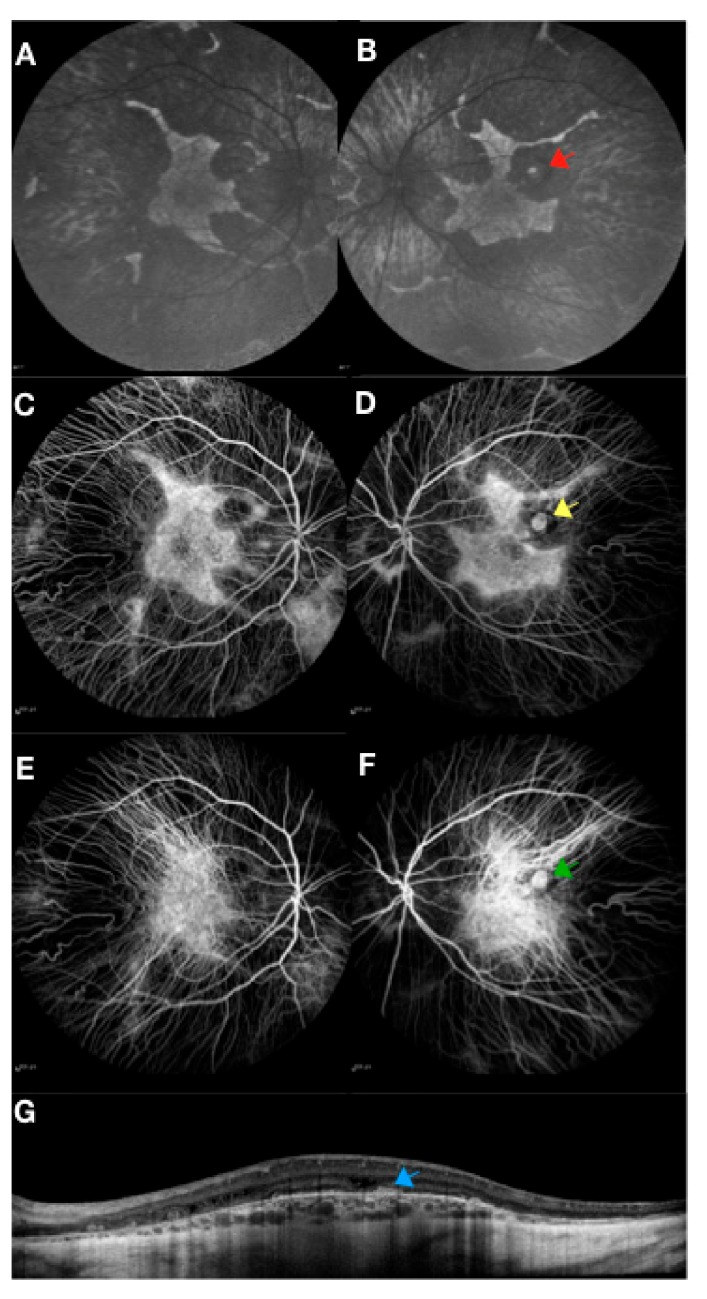
Retinal imaging in a choroideremia patient showing an area of scaring from an old choroidal neovascular membrane in the left eye. Fundus autofluorescence (**A**, **B**), fluorescein angiography (**C**, **D**), indocyanine green angiography (**E**, **F**) and spectral domain optical coherence tomography (**G**) with arrows marking the old scar. Imaging was performed with Heidelberg Spectralis, Heidelberg, Germany.

**Figure 3:**
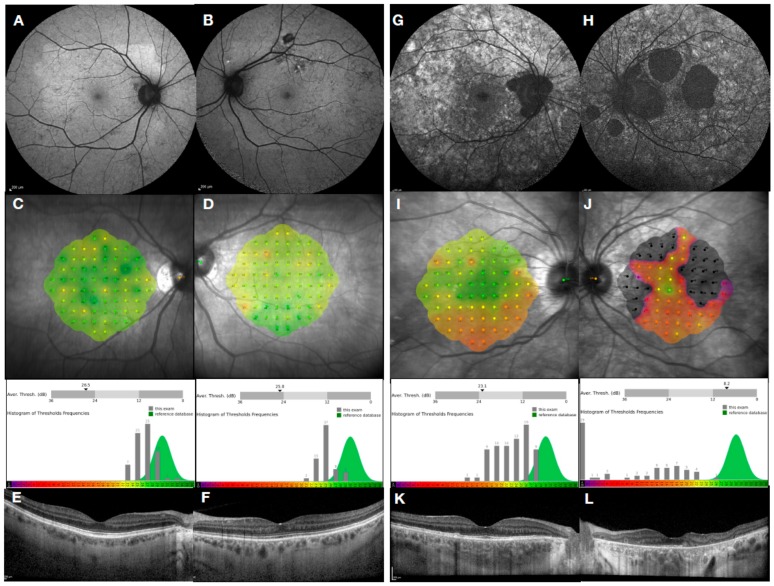
Retinal imaging in two female choroideremia carriers. Phenotype of an asymptomatic mild carrier with Snellen visual acuity of 6/5 in both eyes is shown from (**A**–**F**) and a carrier with a ‘geographic-pattern’ phenotype and reduced visual acuity of 6/7.5 in the right eye and 6/12 in the left eye is shown from (**G**–**L**). Fundus autofluorescence showing very early signs of fine ‘salt and pepper’ mottling (**A**,**B**) compared with coarse mottling and atrophic patches resembling geographic patterns (**G**,**H**). Mesopic microperimetry, MAIA CenterVue SpA, Padova, Italy showing sensitivity maps with corresponding histograms of threshold frequencies. Near-normal central retinal sensitivity is found in mild, asymptomatic carriers (**C**,**D**) compared to reduced retinal sensitivity in affected carriers especially in the left eye of the above case (**I**,**J**). OCT imaging is clinically insignificant in mild, asymptomatic carriers (**E**, **F**) whereas some disruption of retinal pigment epithelium (RPE) and ellipsoid zone is observed in the affected carrier, particularly in the left eye (**K**,**L**).

There is however mounting indirect evidence that the RPE is the primary site of the disease in choroideremia, with the inner (photoreceptor) and outer (choroidal) layers degenerating through secondary mechanisms [5]. The unique pattern of preserved retina and RPE, as seen on autofluorescence imaging (Figure 1), with sharply demarcated edges is very different from many other retinal diseases where preserved regions are more circular or oval. This appearance is, however, almost identical in dominantly inherited *RPE65* retinal diseases. Since *RPE65* is only expressed in the RPE, we know that this phenotype is a feature specific to the RPE (presumably, RPE cell death), giving indirect evidence that choroideremia is a disease driven by RPE loss. The confounding variable in choroideremia is that the REP1 protein is expressed throughout the body [17] and the name ‘choroideremia’ gives the impression that this is primarily a choroidal degeneration. This is not the case, however, because any disease or treatments such as cryotherapy that destroys the RPE layer alone, will eventually lead to secondary atrophy of the underlying choroid, in a similar manner. In other words, choroideremia is the phenotype of complete RPE cell loss. The other relevant factor is that male patients with choroideremia can develop choroidal new vessels (Figure 2) and this clearly shows that the choroidal vasculature has the capacity to regenerate in certain cases. Finally, we know from female carriers (Figure 3) that the pattern of RPE loss is very similar to that in carriers of ocular albinism. There is no evidence of X inactivation leading to patchy loss of the choroid independently in female carriers. 

It is also possible that REP1 expression may be important for rod photoreceptor function [18]. Processing of post-mortem tissue from patients can make histological analyses difficult, and studies using advanced imaging techniques have provided somewhat equivocal results in terms of evidence of independent rod degeneration in humans in areas of the retina where the underlying RPE cells are unaffected by the disease [6,8,18,19,20]. Since patients with choroideremia maintain excellent visual acuity until the very late stages of the disease [6,7,8], it is likely that the REP1 deficiency is not a significant factor for the cone photoreceptors. 

Elucidating the pattern of degeneration in choroideremia may help us understand the basis of the disease and how it progresses [16]. It is not known why the degeneration in choroideremia starts in the equatorial region before spreading anteriorly and posteriorly to reach the macula. The retinal pigment epithelial cell density is roughly similar at 5000 cells per mm^2^ throughout the posterior eyecup. Gyrate atrophy of the choroid however may develop in a similar distribution, although this is in contrast to age-related macular degeneration, which is very much focused in the region around the fovea. In a recent study it was shown that the rate of degeneration in choroideremia followed an exponential decay function and was very similar across patients of different ages [21], but the key factor that determined the severity of the disease was the age of onset of degeneration. It may therefore be possible to predict the severity of the disease simply by measuring the residual area in a patient at a given age, because the progression is likely to be constant in the absence of treatment.

The centripetal degeneration in choroideremia has two phases by fundus autofluorescence-mottled RPE up to the edge and a more central zone of smooth RPE, both of which shrink progressively. In more advanced stages of the disease there is a total loss of smooth zone. The anatomical basis for these two zones is not immediately clear, but it may be that the slightly increased RPE cell density and much thicker choroid at the posterior pole provides some degree of protection against the metabolic stress caused by REP1 deficiency. Recent evidence suggests that there is less preserved autoflourescence area in nasal macula that may be more vulnerable to degeneration [16]. Further studies are necessary to determine whether the RPE zones can predict the health status of the overlying photoreceptors and how these might be affected following treatment. 

## 3. Choroideremia Genotype

The choroideremia gene, *CHM* (OMIM #300390), encodes the REP1 protein, a 653 amino acid polypeptide essential for intracellular trafficking and post-translational prenylation of proteins within the human eye. Currently, there are 346 mutations registered on Leiden Open Variation Database, LOVD^3^ (www.lovd.nl/CHM). Almost all of the identified sequence variations regardless of mechanism, are predicted to be null [3,22,23,24,25]. The mechanisms include insertions and deletions (minor, a few nucleotides, and major involving up to the entire gene length), splice site mutations, missense changes and point mutations that result in stop codons (premature termination codons). Novel mutations have recently been identified involving a deep-intronic region [26] and a promoter region [27] of the *CHM* gene. 

Compared with other genetic diseases including inherited retinal disease, choroideremia has a surprisingly low number of disease-causing missense mutations. This would suggest that the REP1 protein, with 3 principal domains, has no catalytic domains with corresponding mutational hotspots within the gene. This is in contrast to genes that encode enzymes (such as *retinitis pigmentosa GTPase regulator* gene) that typically have such hotspot regions (e.g., ORF15 region). This supports the role of REP1 as a chaperone protein, enhancing activity of another protein, which is important in cell structure and stability. 

Recent evidence shows that the majority of missense mutations are disproportionately found to be single point C to T transitions at C-phosphate-G (CpG) dinucleotides, spread across 5 of only 24 CpG dinucleotides in the entire *CHM* gene [25]. This is consistent with the evolutionary loss of CpG dinucleotides through destabilising methylation and subsequent deamination. Notably, the 5 locations were the only sites at which C to T transitions resulted in a stop codon. Future de novo mutations are likely to arise within these destabilised hotspot loci.

Molecular genetic testing offers means of confirming the clinical diagnosis in choroideremia and is mandatory for the inclusion in gene therapy clinical trials. It also offers a means of identifying carriers and establishing presymptomatic diagnoses in families that carry a pathogenic change. The rate of mutation detection via next generation sequencing has been reported as high as 94% [25]. In cases of unidentified mutations, it is important to request sequencing of the above mentioned deep-intronic and promoter regions, that are not routinely sequenced, to check for pathogenic variations. In addition, functional in-vitro assay that measure levels of REP1 in peripheral blood cells and its prenylation activity [17], can support clinical diagnosis and confirm variants of uncertain pathogenicity. In this regard, choroideremia is different to retinitis pigmentosa, because the unique choroideremia phenotype can justify the additional resources needed to sequence the entire CHM genomic region. 

### 3.1. Genotype–Phenotype Correlation in Choroideremia

Although the clinical phenotype can vary in terms of the age of onset of retinal degeneration and rate of progression, no evidence has been found for genotype–phenotype correlation with regard to onset of symptoms, decline in visual acuity and visual fields [23,24,25], or in the residual retinal area of fundus autofluorescence [25]. The reasons for this are not fully understood, but the lack of correlation may be due to the near universal absence of REP1 irrespective of the causative mutation that range from single point missense changes to whole gene deletions. The phenotypic variation in choroideremia may in part be explained by the degree to which the absence of REP1 can be compensated by other prenylation proteins such as REP2, which shares 95% of its amino acid sequence with REP1 [26,27]. In addition, genetic modifiers and environmental factors may play roles in the onset and progression of degeneration in choroideremia. 

### 3.2. Molecular Mechanisms of Choroideremia

The molecular mechanisms involved in the pathology of choroideremia have recently been reviewed in great detail [5]. However, some basic concepts are worth re-stating and outlining to aid understanding of the disease. The gene that is disrupted in choroideremia produces REP1 protein. Unlike in many other inherited retinal diseases, this protein is not directly involved in the process of phototransduction or in cellular signalling within the retina. Instead, REP1 is a key player in the addition of prenyl groups (prenylation) to the Rab family of GTPases (Rabs). Such hydrophobic prenyl groups are thought to be necessary to anchor Rabs to the membranes of intracellular organelles and vesicles [28].

In the absence of REP1, there is an observable deficit in the prenylation of several different types of Rabs, and their association with membranes appears to be impaired [29]. Because Rabs themselves act as important regulators of intracellular membrane trafficking, many fundamental cellular processes can potentially be impacted by this deficit. Information from a variety of sources points to a deficit in melanosome trafficking, a delay in phagosome degradation and an accelerated accumulation of intracellular deposits in RPE cells caused by loss of REP1 [4,18,30,31,32,33]. The cellular deficits of photoreceptors themselves have been less studied, but it has been suggested that there is mislocalisation of opsin and shortening of photoreceptor outer segments in mice that is independent of RPE degeneration [4].

Fortunately, the absence of REP1 does not appear to be catastrophic for all human cells, which is likely due to the fact that there is a built-in redundancy in this system, provided by the presence of the *CHML* gene [34,35]. The *CHML* gene is thought to be an autosomal retrogene of *CHM*, created by the reverse transcription of the mRNA of the original gene and reinsertion in a new genomic location that occurred sometime during vertebrate evolution. The protein product of *CHML*, known as REP2, appears to be able to largely compensate for the loss of REP1. Although a prenylation deficit of certain Rabs can be detected in several cell types of the body [29,36,37], a single report of a systemic, blood-related, clinical phenotype have not been substantiated [38,39] and loss of REP1 appears to cause cellular dysfunction and death that is limited to specific ocular tissues and manifest as a specific disease of the retina. Differential spatial expression does not provide an obvious answer, as both REP1 and REP2 are expressed ubiquitously. 

In truth, the reason why absence of REP1 drives a specific degeneration of the RPE and photoreceptor cells remains a mystery. Perhaps more than other cell types, RPE and photoreceptor cells require acute and sensitive regulation of intracellular membrane trafficking to fulfil their cellular functions. Combined with the fact that there is not any appreciable post-natal replacement of these cells, it may simply be that these cell types are sensitive to the generalised, ongoing prenylation deficit, become ‘worn-out’ early than usual, and undergo a type of accelerated aging and cell death. Alternatively, it has been proposed that REP1 has a selective affinity to particular Rabs that are of special significance to the cell types affected in the disease. For example, it has been suggested there is a particular requirement for correctly prenylated Rab27a to mediate melanosome trafficking in RPE cells [29,40] and Rab6, 8 and 11 might be important in targeting rhodopsin-bearing vesicles to the photoreceptor outer segment [41,42]. Biochemical assays have suggested that REP1and REP2 have largely overlapping substrate specificities but differences in the association with other catalytic units within the prenylation process might contribute instead [43,44,45].

### 3.3. Gene Therapy for Choroideremia

Gene based therapies show great promise for the treatment of inherited retinal disease, including choroideremia [46]. Recent advances have paved a successful progression of gene therapy clinical trials on choroideremia (Table 1). The first phase I/II trial started in Oxford, UK in 2011, using a subretinal delivery of AAV2-REP1 in 14 male patients with choroideremia [9,10]. The two-year trial results were recently reported [11] with median gains in visual acuity (measured by Early Treatment Diabetic Retinopathy Study, ETDRS chart) of 4.5 letters in treated eyes versus 1.5 letter loss in untreated eyes across the cohort at 24 months post treatment. Six treated eyes gained more than 5 ETDRS letters. In two patients with the greatest gains in visual acuity, improvements were noted by 6 months post treatment, and sustained at up to 5 years of follow-up. Two patients in the cohort had complications, one related to surgery (retinal overstretch and incomplete vector dosing) and the other had postoperative inflammation. Both of these events resulted in protocol changes which included developing an automated subretinal injection system and a more prolonged post-operative immunosuppressive regimen.

These encouraging safety and efficacy signals prompted additional trials using the same vector (sponsored by Nightstar Therapeutics, UK) at other international sites including Canada (NCT02077361), USA (NCT02553135) and Germany (NCT02671539) all reporting similar results [12,13,14], following which a phase III trial started in 2017 at multiple international sites. Independent to the Nightstar led trials, another phase I/II trial (NCT02341807) using a similar AAV vector construct (without the woodchuck hepatitis virus posttranscriptional regulatory element) begun in 2015 in Philadelphia, USA. The results of this trial are expected in the coming years. 

The above-mentioned early phase I/II gene therapy clinical trials recruited patients with advanced disease with early efficacy signals suggesting that vision can be restored following treatment. Reassuring safety data, following improvements in the surgical technique, prompted initiation of a phase II trial (NCT02407678) sponsored by University of Oxford that included patients with early central degeneration and normal visual acuity. The REGENERATE trial recently completed recruitment of 30 male patients with choroideremia with prediction that earlier intervention might slow down or halt the degeneration prior to irreversible structural disorganisation.

The solstice study is an observational, long-term follow up study of 100 participants that will evaluate the safety and efficacy of the AAV2-REP1 used in the above-mentioned interventional choroideremia trials. 

The outcomes of clinical trials are measured in terms of clearly defined clinical endpoints, which predict the success and ultimately the approval of new treatments. These outcomes must be selected carefully to capture the most sensitive and reliable measures of the disease progression during the course of a clinical trial and will critically depend on the stage of retinal degeneration. In the reported choroideremia trials, the primary endpoint was the change from baseline in best-corrected visual acuity (BCVA) in the treated eye compared to the untreated eye with evidence of gains in vision after gene therapy in treated eyes. This suggests that BCVA can be used as a viable primary outcome in cases of advanced choroideremia, where disease process has already affected the visual acuity. Indeed, the phase III STAR trial is using BCVA as a primary outcome measure. However, in patients with early disease stage with near-normal vision, BCVA may not be the most sensitive outcome measure, especially since the visual loss in choroideremia typically progresses very slowly. Thus, for the REGENERATE trial, secondary endpoints including the measure of central visual field by microperimetry and anatomical measures such as fundus autofluorescence and optical coherence tomography may prove to be additional valuable outcomes. However, measurements of these secondary outcomes may not always be straightforward, and need to be interpreted with caution. For example, the remaining autofluorescence area may not be easily demarcated, even with the use of automated algorithms, which may influence area measurements especially following sub-retinal gene therapy which may differentially affect central (para-foveal) and peripheral areas of the treated island. 

## 4. Should We Treat Female Carriers in the Future?

Heterozygous female choroideremia carriers often show generalized RPE mottling due to random X-inactivation (Figure 3A–F) and are usually asymptomatic or show early deficits in dark adaptation. In some carriers a coarser pattern of degeneration is seen, with patches of atrophy interspersed with normal tissue (Figure 3G–L). Usually, a mild reduction in retinal function is observed with this carrier phenotype. Occasionally, however, female carriers manifest with more severe male-like pattern of retinal degeneration with associated deficit in visual function [47]. This is most likely the result of skewed X-inactivation, or the proportion of cells expressing the mutant X chromosome, which occurs during early retinal development. 

Choroideremia gene therapy trials are currently including affected male subjects only. For the majority of female carriers who are mildly affected and asymptomatic or have minor deficits in night vision or visual fields, treatment may not be necessary. Such functional deficits are usually slowly progressing with the majority of cases being able to maintain driving standard vision. However, the more severe female carrier phenotypes, with associated visual field loss and reduction in visual acuity, are likely to benefit from gene therapy and could be included in future clinical trials. Careful characterisation and geneotype-phenotypes correlations will help with the inclusion criteria and give insight into the optimal timing for successful gene therapy. 

## 5. Alternative Therapies

The potential therapy that has been discussed in this review is gene replacement/augmentation therapy. This is the therapy that has advanced the furthest clinically but there are other potential therapies worth considering.

Instead of adding a working copy of the *CHM* gene, it may instead be possible to alter the patient’s own copy with gene editing. Techniques to achieve this, such as zinc finger nucleases or Tal-effector nucleases (TALENs), have existed for some time, but the clinical relevance of these techniques has been somewhat limited by the low editing efficiencies generally achieved. The development of CRISPR/Cas (clustered regularly interspaced short palindromic repeats/CRISPR-associated nuclease 9) technology has given gene editing a renaissance for two reasons. Firstly, gene-editing efficiency appears to be generally better, with the potential to be more clinically meaningful. Secondly, in the CRISPR/Cas9 system, most of the investigational medicinal product can remain the same and only a specific RNA guide sequence needs to be developed to target a site within the disease specific gene—this is more attractive in terms of a clinical development pathway. Gene editing therapy is most useful when there is a need to correct or silence a mutated gene, such as when a missense mutation leads to production of dominant negative or toxic gain-of-function protein, which normally manifests as autosomal dominant and semi-dominant disease [48]. Because the vast majority of mutations in choroideremia are effectively null and therefore result in no detectable protein [24] there is no compelling need to develop a gene editing approach, and simply adding a correct copy as an episomal transgene would be sufficient to result in a therapeutic effect. Correcting the genomic copy of the gene might provide higher confidence of a correct and sustained level of expression, given that the gene would be subject to regulation by its normal transcriptional regulation and epigenetic environment. However, there is evidence that expression from a transgene can be sustained for years when using the appropriate delivery vector and expression cassette [49]. For choroideremia, there is no cell type in which ectopic expression may be predicted to cause a problem, as the protein in normally ubiquitously expressed. In terms of the level of expression, we know that the level of restored REP1 expression is inversely proportional to the prenylation deficit, and so far there is no evidence of overexpression causing toxicity [50]. Although it may be theoretically possible to develop a gene editing approach for some mutations that cause choroideremia, using CRISPR/Cas9, the effectiveness of such strategies has not yet been well established in the retina. Therefore, as gene editing might offer only marginal benefits over gene replacement, it is not currently an attractive strategy of treating choroideremia.

Another therapy that has been suggested and developed is the use of drug-stimulated translational read-through (RT) of premature termination codons (PTC). Nonsense mutations arise when a point mutation converts an amino-acid codon into a PTC that can cause premature translational termination of the mRNA, and subsequently inhibit normal full-length protein expression. Occasionally, instead of translational termination, read-through occurs. Here, a partial mispairing of codon–anticodon is successful, an amino acid is incorporated and protein synthesis continues. Small molecule translational read-through inducing drugs (TRIDs) exist that form the basis of the proposed therapy [51]. Nonsense mutations are the cause of choroideremia in over 30% of patients [52], so, although this will not be appropriate for all patients, there is a significant proportion in which it might be used.

Translational read-through inducing drugs have been used in clinical trials for life-limiting congenital diseases, such as Duchenne muscular dystrophy (DMD) and cystic fibrosis. Early trials appeared to successfully suppress premature stop mutations in patients, but there were concerns over toxicity and the need for repeated intramuscular or intravenous dosing. A newer read-through drug, Ataluren (PTC124), showed a good safety profile when administered orally and the clinical benefit shown in DMD has led to its approval in the EU for this disease [53]. Although no adverse effects have been observed so far, even the approximately 48 weeks of administration given in the clinical studies do not approach the decades of treatment that would be necessary for choroideremia. Preclinical work in the lower-vertebrate, zebrafish model has been important in developing the proof-of-concept, as this is currently the only existing model of choroideremia with a nonsense mutation [54,55]. However, absence of the CHML (REP2) gene in zebrafish means that the CHM mutation is lethal––translational read-through inducing drugs increase the lifespan of the zebrafish model but this is outcome is not directly clinically relevant. The ability of TRIDs to rescue the Rab prenylation defect in fibroblast of a patient with a particular choroideremia nonsense mutation is encouraging, despite the fact that levels of full-length REP1 protein remained below the level of detection [55]. Given the relatively slow disease progression and the potential risks and cost to the patient from long-term administration of TRIDs, it would be judicious to establish that the correction of the prenylation deficit by TRIDs is present in fibroblast from patients with the equivalent nonsense mutations in which treatment will be attempted in any clinical study [56].

It might be argued that systemic or ocular administration of TRIDs has the potential to treat a larger area of retina when compared to gene therapy, as the former might spread by local diffusion while the latter is limited by the extent of the subretinal bleb. However, to our knowledge, the local concentration achieved in the posterior segment of the eye has never been measured when TRIDs are taken orally or administered locally. The effect of TRIDs appears to often follow an inverted u-shaped dose-response curve, so the pharmacokinetics of therapy may be critical important [57]. Until such questions are addressed, it would appear that translational read-through inducing drugs do not represent a superior strategy compared to gene replacement therapy.

Recent work has identified that antisense oligonucleotides (AONs) may also provide another potential therapy for choroideremia [58]. In some cases of choroideremia, deep-intronic mutations can create a cryptic splice acceptor site that results in the insertion of a pseudoexon in the *CHM* transcript. This disrupts gene function, and specific AONs can be designed to bind to the pre-mRNA and redirect the splicing process, potentially returning it to a normal, working transcript [59,60]. For choroideremia, AONs therapy has shown some promising in vitro results but is further along the clinical development pathway for several inherited disorders, including other forms of inherited retinal dystrophy [59,60]. As AON therapy relies on particular types of mutations, it will not be relevant for all cases of choroideremia and such a strategy is most attractive when conventional gene replacement therapy is not possible because of the large size of the coding sequence of the genes involved, such as in *CEP290*-associated Leber congenital amaurosis [59,60].

The therapies above aim to slow down or stop the degeneration of the retina and RPE and are obviously the preferred choice. However, it is also worth considering strategies that might restore vision in the late stages of the disease, when the majority of photoreceptors have already been lost. Cell transplantation is an interesting strategy for the treatment of inherited retinal disease, but this might present a significant challenge in late-stage choroideremia, where RPE and choroid have been lost along with the degenerating photoreceptors. A more feasible approach may be to use some form of retinal prosthesis. Although most systems rely on surviving inner retinal layers, with intact ganglion cell nerve conduction, there is no dependence on survival of the RPE, photoreceptors or choroid. The Argus II retinal prosthesis, an epiretinal device approved for commercial use in advanced retinal degeneration in the EU and USA, has been implanted in at least one patient with choroideremia [61]. This device has a very good safety profile and various improvements in visual function have been reported, although these vary widely between individuals [62]. Other devices exist or are in development (44-channel suprachoriodal Bionic Eye Device (NCT03406416) Melbourne, Australia and Intelligent Retinal Implant System, IRIS V1 (NCT01864486) and V2 (NCT02670980) Pixium Vision SA) that could theoretically restore much greater levels of visual function than the Argus II, however, stopping cell loss, even at a late-stage will likely still result in a better functional outcome. Another potential therapy to restore vision in choroideremia is to render the remaining cells of the retina sensitive to light by ectopically expressing light-sensitive ion channels or opsins. This strategy, known as optogenetics, has its own considerations and challenges, which will not be discussed extensively here. Suffice to say, a number of systems are in various stages of pre-clinical development and are beginning to be investigated in clinical trials [63,64,65]. Again, the level of vision that can be restored by this method is likely to be relatively crude, however, this is likely to be comparable to any retinal prosthesis and may offer specific benefits such as less invasive surgery and potential restoration of a wider visual field.

## 6. Summary

Molecular mechanisms in choroideremia are well established. Ultimately, the absence or reduced prenylation of REP1 activity disrupts intracellular trafficking pathways leading to accumulation of toxic products and premature degeneration of the retina and vision less. Logically then, replacement of REP1 to the retinal tissue, via gene-based therapy, could restore cellular function and slow down the degeneration. Multiple clinical trials are underway testing this hypothesis. The trials are using subretinal delivery of AAV2-REP1 to target surviving central islands of the retina with promising safety and early efficacy results. 

Despite ubiquitous expression of REP1, a robust systemic association with choroideremia has not been identified, although the prenylation defect is visible in assays of the peripheral blood cells. This assay can be used to support the diagnosis of choroideremia. It is not known why the retina is the only part of the body that becomes clinically affected by the lack of REP1 activity. Moreover, the complex interactions between different retinal cell types during the pathogenesis of choroideremia mean that it is difficult to deconvolve the exact order in which RPE, photoreceptors and the choroid degenerate. It appears likely that the RPE is directly affected by the loss of REP1, and is a key driver of pathogenesis, but the importance of primary or secondary degeneration of photoreceptors is less clear. Elucidating these mechanisms may help us to understand what triggers the onset of clinically significant degeneration and how the rate of degeneration in each cell type might be affected following treatment.

Evidence to date has shown no apparent genotype–phenotype correlation within the spectrum of reported CHM mutations, with regard to the onset of symptoms and the rate of functional visual decline. Since variations in male phenotypes cannot be explained by mutations in *CHM* only, genetic modifiers or environmental factors must play a role in the onset and progression of degeneration in choroideremia. Ongoing natural history studies are adding insight into the progression of the disease and the characteristics of the clinical phenotype that will help to establish the optimal therapeutic window for choroideremia. Female carriers should be enrolled into natural history studies with aim to offer gene therapy (under the realm of clinical trials) to those affected by skewed X inactivation.

## Figures and Tables

**Table 1:** Summary of interventional gene therapy clinical trials in choroideremia.

Clinical trial	Intervention	Clinical centre	References
Phase I/II NCT01461213 Start date: 2011 Completed 2018	Gene therapy involving subretinal delivery of AAV2-REP1	University of Oxford, UK	Lancet, 2014 [9] NEJM, 2016 [10] Nat Med, 2018 [11]
Phase I/II NCT02341807 Start date: 2015 Ongoing	Gene therapy involving subretinal delivery of AAV2-REP1	Philadelphia, USA Spark Therapeutics	No reports to date
Phase I/II NCT02077361 Start date: 2015 Completed 2018	Gene therapy involving subretinal delivery of AAV2-REP1	University of Alberta, Canada	Am J Ophthalmol, 2018 [12]
Phase II NCT02553135 Start date: 2015 Completed 2019	Gene therapy involving subretinal delivery of AAV2-REP1	University of Miami, USA	Am J Ophthalmol, 2019 [13]
Phase IINCT02671539 THOR TRIAL Start date: 2016 Completed 2018	Gene therapy involving subretinal delivery of AAV2-REP1	University of Tubingen, Germany	Retina, 2018 [14]
Phase II NCT02407678 REGENERATE TRIAL Start date: 2016 Ongoing	Gene therapy involving subretinal delivery of AAV2-REP1	University of Oxford and Moorfields Eye Hospital, UK	No reports to date
Phase II NCT03507686 GEMINI TRIAL Start date: 2017 Ongoing	Gene therapy involving bilateral subretinal delivery of AAV2-REP1	Nightstar Therapeutics (now Biogen) International, Multi-centre	No reports to date
Phase III NCT03496012 STAR TRIAL Start date: 2017 Ongoing	Gene therapy involving subretinal delivery of AAV2-REP1	Nightstar Therapeutics (now Biogen), International, Multi-centre	No reports to date
Observational NCT03584165 SOLSTICE TRIAL Start date: 2018 Ongoing	Long-term follow up study evaluating the safety and efficacy of AAV2-REP1 used in antecedent interventional choroideremia studies, 100 participants	Nightstar Therapeutics (now Biogen), International, Multi-centre	No reports to date
